# HDAC7‐mediated control of tumour microenvironment maintains proliferative and stemness competence of human mammary epithelial cells

**DOI:** 10.1002/1878-0261.12503

**Published:** 2019-06-27

**Authors:** Valentina Cutano, Eros Di Giorgio, Martina Minisini, Raffaella Picco, Emiliano Dalla, Claudio Brancolini

**Affiliations:** ^1^ Department of Medicine Università degli Studi di Udine Italy

**Keywords:** breast cancer, CRISPR/Cas9, HDAC7, IL24, MEF2, RAS

## Abstract

HDAC7 is a pleiotropic transcriptional coregulator that controls different cellular fates. Here, we demonstrate that in human mammary epithelial cells, HDAC7 sustains cell proliferation and favours a population of stem‐like cells, by maintaining a proficient microenvironment. In particular, HDAC7 represses a repertoire of cytokines and other environmental factors, including elements of the insulin‐like growth factor signalling pathway, IGFBP6 and IGFBP7. This HDAC7‐regulated secretome signature predicts negative prognosis for luminal A breast cancers. ChIP‐seq experiments revealed that HDAC7 binds locally to the genome, more frequently distal from the transcription start site. HDAC7 can colocalize with H3K27‐acetylated domains and its deletion further increases H3K27ac at transcriptionally active regions. HDAC7 levels are increased in RAS‐transformed cells, in which this protein was required not only for proliferation and cancer stem‐like cell growth, but also for invasive features. We show that an important direct target of HDAC7 is *IL24*, which is sufficient to suppress the growth of cancer stem‐like cells.

**Abbreviations**
4‐OHT: 4‐hydroxytamoxifen
BrdU: bromodeoxyuridine
ER: oestrogen receptor
GSEA: gene set enrichment analysis
H3K27ac: histone H3 lysine 27 acetylation
IGF: insulin‐like growth factor
KO: knock‐out
MM: mammosphere medium
MSigDB: Molecular Signatures Database
TSS: transcription start site

## 1 Introduction

Environmental adaptations require fine‐tuning of gene transcription supervised by chromatin modifications. A repertoire of epigenetic regulators controls the milieu of the epigenetic marks that influence chromatin compaction or opening (Vineis *et al*., 2017). Among transcriptional repressors, class IIa HDACs are important regulators of genetic programmes, which orchestrate different cellular fates. This family of enzymes includes four members: HDAC4, HDAC5, HDAC7 and HDAC9, which are subjected to environmental regulated nuclear/cytoplasmic shuttling (Clocchiatti *et al*., 2011; Di Giorgio and Brancolini, 2016). In vertebrates, class IIa HDACs show neglectable lysine‐deacetylase activity; however, through the recruitment of the N‐CoR/SMRT/HDAC3 complex, they can coordinate and buffer histones acetylation (Desravines *et al*., 2017; Lahm *et al*., 2007). An extended amino‐terminal region, devoted to the interaction with other corepressors and different transcription factors, grants selective genomic activities to these epigenetic regulators (Clocchiatti *et al*., 2011; Di Giorgio and Brancolini, 2016).

The ability of class IIa HDACs to act as platforms for the recruitment of different partners renders these coregulators highly tuneable in terms of genes modulated and consequent biological responses. This plasticity holds remarkably true for HDAC7. Genetic studies in mice have proved that *Hdac7* controls vascular stability and remodelling (Chang *et al*., 2006), as well as B‐cell and T‐cell development (Azagra *et al*., 2016; Kasler and Verdin, 2007; Navarro *et al*., 2011).

Epigenetic plasticity is emerging as an important hallmark of cancer (Barneda‐Zahonero and Parra, 2012; Feinberg *et al*., 2016; Flavahan *et al*., 2017; Koschmann *et al*., 2017). Up‐regulation of HDAC7 expression in rodent and human cells can cooperate for the neoplastic transformation (Di Giorgio *et al*., 2013; Lei *et al*., 2017; Paluvai *et al*., 2018; Rad *et al*., 2010). In breast and ovary cancers, HDAC7 is highly expressed in cancer stem cells and it is involved in stem cell maintenance (Witt *et al*., 2017). Although there are evidences for a role of HDAC7 in the neoplastic transformation, the genetic and epigenetic changes under HDAC7 supervision are not fully elucidated. To achieve this goal, we knocked out HDAC7 in human mammary epithelial cells MCF10A, which offer the opportunity of studying proliferation, stemness and oncogenesis (Amin *et al*., 2016; Liu *et al*., 2013; Qu *et al*., 2015).

## 2 Materials and methods

### 2.1 Cell cultures and reagents

MCF10A cells were grown as previously described (Clocchiatti *et al*., 2015). HEK‐293T, AMPHO and SKBR3 cells were grown in Dulbecco's modified Eagle's medium (DMEM; Euroclone, Milan, Italy) supplemented with 10% FBS (Euroclone). Cells expressing the inducible form of HDAC7 were grown in complete F12/DMEM medium without phenol red (Sigma‐Aldrich, St. Louis, MO, USA) and with 5% charcoal‐stripped horse serum. 4‐hydroxytamoxifen (4‐OHT; Sigma‐Aldrich) was used at 1 μm.

### 2.2 Three‐dimensional morphogenetic and mammosphere assays

The 3D morphogenetic assay was conducted as previously described (Clocchiatti *et al*., 2015). Cells (3 × 10^4^) were plated in a thick layer of ~ 1–2 mm of laminin‐rich extracellular matrix (ECM; Cultrex‐Sigma‐Aldrich). Mammospheres were grown in mammosphere medium (MM) constituted by Ham's F12/DMEM 1 : 1 medium (Sigma‐Aldrich), supplemented with B27 (1×) (Gibco, Waltham, MA, USA) and EGF (20 ng·mL^−1^) (Peprotech, London, UK). For MCF10/RAS cells, 0.5% (final concentration) of methylcellulose was added to the MM. Before seeding, cells were stained with trypan blue and only healthy cells were counted. 1 × 10^3^ cells were seeded on Ultra‐Low Attachment multiwells (Corning, New York, NY, USA) and cultivated for 10 days. For BMP4 (Thermo Fisher, Waltham, MA, USA) and IL24 (Peprotech) treatments, MCF10A were pretreated for 2 days. Spheres over 50 μm of diameter were counted. Conditioned medium was obtained by growing MCF10A cells in complete MM for 2 days. Next, medium was collected, filtered and diluted 1 : 1 with fresh new MM and used for the experiments. Images were collected by using a Leica AF 6000LX microscope (Leica Microsystems, Mannheim, Germany). Area was measured with imagej software (NIH, Bethesda, MD, USA).

### 2.3 Generation of MCF10A/HDAC7^−/−^ cells

CRISPR/Cas9 technology was used as previously described (Di Giorgio *et al*., 2017). Clones were screened by PCR and immunoblot. Sanger sequencing was applied for the final validation. gRNAs were designed using ‘CRISPR design’ available from http://crispr.mit.edu/ gRNA exon 3: 5′ CACCGAGCGCTCGGTGGAGCCCATG 3′; gRNA exon 4: 5′ CACCGCCGATGCCCGAGTTGCAGG 3′; gRNA exon 5: 5′ CACCGGGTCAAGCAGAAGCTACGG 3′ cloned into the lentiviral plasmid pLentiV2 which expresses the Cas9 gene. CTRL cells were infected with the pLentiV2 lacking the guide.

### 2.4 Immunofluorescence and immunoblotting

Cells were fixed with 3% paraformaldehyde and permeabilized with 0.1% Triton X‐100. The secondary antibodies were Alexa Fluor 488‐, 546‐ or 633‐conjugated anti‐mouse and anti‐rabbit secondary antibodies (Molecular Probes, Eugene, OR, USA). Actin was labelled with phalloidin‐AF546 (Molecular Probes). Cells were imaged with a Leica confocal scanner SP2. Nuclei were stained with Topro‐3 (Life Technologies, Waltham, MA, USA) or Hoechst 33258 (Sigma‐Aldrich).

Cell lysates after SDS/PAGE and immunoblotting were incubated with primary antibodies. Secondary antibodies were obtained from Sigma‐Aldrich, and blots were developed with SuperSignal West Dura (Pierce, Waltham, MA, USA).

### 2.5 Antibodies

The primary antibodies used were as follows: MEF2D (BD Biosciences, San Jose, CA, USA); MEF2A (C‐21 Santa Cruz Biotechnology, Dallas, TX, USA); p53 and pERK (Cell Signaling Technology, Leiden, the Netherlands); p21, Actin, anti‐BrdU, FLAG M2 and RACK‐1 (Sigma‐Aldrich); RAS (Abcam, Cambridge, MA, USA); GFP and HDAC4 (Paroni *et al*., 2004); and HDAC5 (Clocchiatti *et al*., 2015). Anti‐CD44‐FITC (BD Biosciences) and CD24‐APC (BioLegend, San Diego, CA, USA) were used with matched control antibodies, anti‐mouse IgG1 FITC and IgG2a APC (Miltenyi Biotec, Bergisch Gladbach, Germany). HDAC7 antibodies were generated in rabbits by injecting recombinant histidine‐tagged HDAC7 fragment aa 261–522. For anti‐HDAC7 antibody purification, HDAC7 was fused to glutathione *S*‐transferase and cross‐linked to glutathione‐Sepharose as described previously (Paroni *et al*., 2004).

### 2.6 Plasmid construction, transfection, retroviral and lentiviral infection, siRNA delivery

To generate pWZL‐Hygro‐HDAC7 WT‐ER, an *Eco*RI fragment of HDAC7 WT was cloned into pWZL‐Hygro‐ER. Cells expressing HDAC7‐ER, RAS‐G12V, MEF2‐VP16 and MEF2ΔDBD transgenes were generated by retroviral infection as described previously (Cernotta *et al*., 2011).

### 2.7 RNA extraction and quantitative qRT/PCR

Cells were lysed using TRI Reagent (Molecular Research Center, Cincinnati, OH USA). About 1.0 μg of total RNA was retro‐transcribed by using 100 units of M‐MLV Reverse Transcriptase (Life Technologies). qRT/PCRs were performed using SYBR green technology (KAPA Biosystems, Wilmington, MA, USA). Data were analysed by comparative threshold cycle using HPRT as normalizer.

### 2.8 RNA expression array and data analysis

Aliquots of RNAs, purified using RNeasy columns (Qiagen, Hilden, Germany), were amplified according to the specifications of the Illumina TotalPrep RNA Amplification Kit (Ambion, Waltham, MA, USA). Hybridization on Illumina whole‐genome HumanHT‐12 v 4.0 chip (Illumina, San Diego, CA, USA), scanning and background subtraction were done according to the manufacturer's specification. Fold‐change and *P*‐values for each probe set were calculated using a moderated *t*‐statistic in the limma package (Ritchie *et al*., 2015), with the variance estimate being adjusted by incorporating global variation measures for the complete set of probes on the array. *P*‐values were then corrected for multiple hypothesis testing using the Benjamini and Hochberg methods. In order to select genes that are robust HDAC7 targets, we assumed that *HDAC7* ^*+/+*^ and *HDAC7* ^*−/−*^ */HDAC7‐ER* samples could be considered as replicates, similarly to *HDAC7* ^*−/−*^ and *HDAC7* ^*−/−*^ */ER*. Differentially expressed genes were selected for fold changes |> 1.5| and *P* values < 0.05. Gene set enrichment analysis (GSEA) and the MSigDB database http://software.broadinstitute.org/gsea/index.jsp (Liberzon *et al*., 2015; Subramanian *et al*., 2005) were used to investigate statistically significant functional associations.

### 2.9 Cell cycle FACS analysis, BrdU and transwell migration assays

For cytofluorimetric analysis, cells were fixed with ethanol (O/N), treated with RNase A (AppliChem Lifescience, Darmstadt, Germany) and stained with 10 μg of propidium iodide (Sigma‐Aldrich). For S‐phase analysis, cells were grown for 3 h with 50 μm bromodeoxyuridine (BrdU; Sigma‐Aldrich). After fixation, coverslips were treated with HCl and processed for immunofluorescence. Invasion assay was performed as previously described (Di Giorgio *et al*., 2017). As chemoattractant, complete DMEM/F12 with EGF was added in each lower chamber. DMEM/F12 without EGF was used to evaluate random invasion.

### 2.10 ChIP, library construction, ChIP‐seq and NGS data analysis

ChIP was performed as previously described (Di Giorgio *et al*., 2017), with some modifications. In brief, 50 μg of chromatin was immunoprecipitated with 2 μg of anti‐H3K27ac (Abcam; ab4729), 6 μg of anti‐HDAC7 antibody or control IgG. After RNAseA treatment (Ambion) and de‐crosslinking, DNA was purified with Zymo ChIP columns. Three independent experiments were pulled, and 5 ng of total DNA was used to prepare ChIP‐seq libraries, according to TruSeq ChIP Sample Preparation guide (Illumina). Libraries were sequenced on the Illumina HiSeq 2000 sequencer. The quality of sequencing reads was evaluated using FastQC. Sequencing reads from ChIP‐seq experiments were aligned to the NCBI *GRCh38* human reference with bowtie 2 (Langmead and Salzberg, 2012). Peak calling was performed against input sequences using the homer software (Heinz *et al*., 2010). Peak heatmaps, gene annotations, Venn diagrams and bar plots representing the peak localization in genomic elements/distance from transcription start site (TSS) were obtained using the ChIPseeker R/Bioconductor package (Yu *et al*., 2015).

### 2.11 Statistics

Results were expressed as means ± standard deviations for at least three independent experiments. Statistical analysis was performed using Student's *t* test with the level of significance set at *P* < 0.05. *P < 0.05; **P < 0.01; ***P < 0.005. Data from 3D acinar area and mammosphere formation assays were analysed using the one‐way ANOVA (and nonparametric) test (prism graphpad software; GraphPad software, La Jolla, CA, USA).

## 3 Results

### 3.1 HDAC7 influences proliferation of mammary epithelial cells

**Figure 1:**
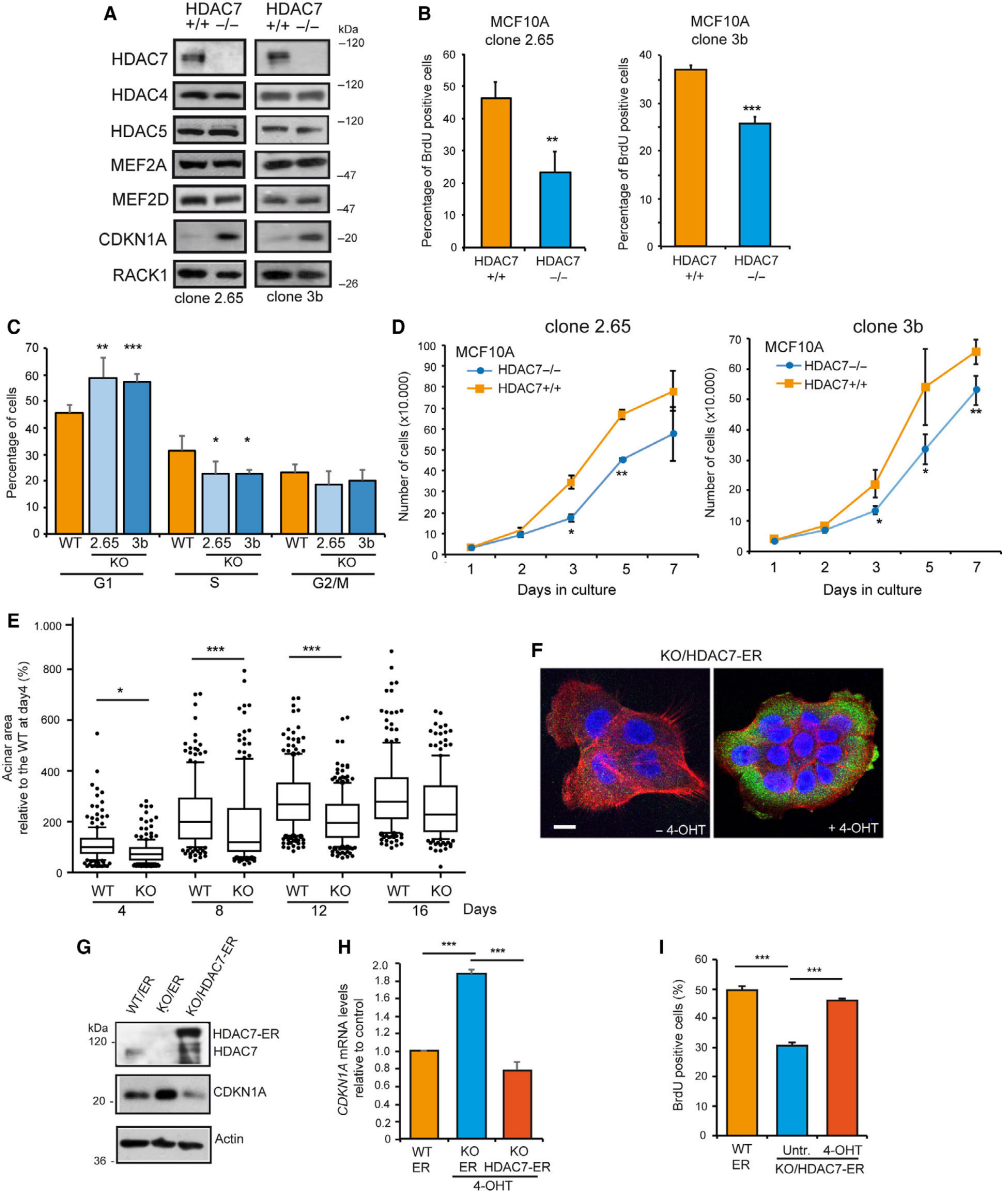
Generation and characterization of MCF10A HDAC7^*−/−*^ cell lines. (A) Immunoblot analysis of class IIa HDACs, MEF2 family members and CDKN1A levels in different clones of *HDAC7* ^*−/−*^ and *HDAC7* ^*+/+*^ MCF10A cells. RACK1 was used as loading control. (B) S‐phase determination by BrdU incorporation in *HDAC7* ^*+/+*^ and *HDAC7* ^*−/−*^ MCF10A clones. After 24 h from seeding, BrdU was added for 3 h. Data are presented as mean ± SD (*n* = 3). We marked with **P(Kruskal–Wallis) < 0.01, ***P(Student) < 0.005. (C) Cell cycle profiles by cytofluorimetric analysis of *HDAC7* ^*+/+*^ (WT) and *HDAC7* ^*−/−*^ (KO) cells (clone 2.65 and clone 3b). Data are presented as mean ± SD (*n* = 3). We marked with *P(Kruskal–Wallis) < 0.05, **P < 0.01, ***P < 0.005. (D) Cell growth assay of the indicated MCF10A cell lines. Only trypan blue‐negative cells were counted. Data are presented as mean ± SD (*n* = 3). We marked with *P(Student) < 0.05, **P(Student) < 0.01. (E) Tukey box‐plots illustrating acinar sizes after culturing MCF10A/*HDAC7* ^*+/+*^ (WT) and (KO) MCF10A/*HDAC7* ^*−/−*^ (clone 3b) cells, in 3D conditions for the indicated days (*n* = 4). We marked with *P(Dunn) < 0.05, ***P(Dunn) < 0.005. (F) Confocal pictures of MCF10A/*HDAC7* ^*−/−*^ cells expressing HDAC7‐ER treated or not with 4‐OHT. After fixation, immunofluorescences were performed to visualize HDAC7 using a specific antibody (green) and F‐actin with AF546‐phalloidin (red). Nuclei were stained with Topro‐3 (blue). Images are shown in pseudocolors. Scale bar, 50 μm. (G) Immunoblot analysis of HDAC7 and CDKN1A levels in the indicated MCF10A cells expressing HDAC7‐ER or ER and treated with 4‐OHT. Actin was used as loading control. (H) mRNA expression levels of *CDKN1A* as measured by qRT/PCR in the indicated MCF10A cells expressing HDAC7‐ER or ER and treated with 4‐OHT for 36 h. Data are presented as mean ± SD (*n* = 4). We marked with ***P(Kruskal–Wallis) < 0.005. (I) S‐phase determination by BrdU incorporation in the indicated MCF10A cells expressing HDAC7‐ER or ER. Data are presented as mean ± SD (*n* = 4). We marked with ***P(Kruskal–Wallis) < 0.005.

The CRISPR/Cas9 technology was used to generate *HDAC7* ^*−/−*^ MCF10A mammary epithelial cells. We characterized in parallel two different clones generated by two different pairs of gRNAs (Fig. S1A). HDAC7 abrogation does not trigger compensatory feedbacks at the levels of other class IIa HDACs and MEF2 family members expressed in MCF10A cells (Fig. 1A). HDAC9, MEF2B and MEF2C are expressed at very low levels (almost undetectable) in this cell line. Instead, the expression of the CDK inhibitor *CDKN1A* was increased. Accordingly, the percentage of cells replicating the DNA was reduced in *HDAC7* ^*−/−*^ compared to *HDAC7* ^*+/+*^ cells (Fig. 1B,C). Cell cycle analysis evidenced that *HDAC7* ^*−/−*^ cells show a prolonged G1 phase (Fig. 1C), with a consequent growth reduction (Fig. 1D). This proliferative defect was maintained in the 3D culture system (Clocchiatti *et al*., 2015), where the acinar size was significantly reduced at days 4, 8 and 12 in the absence of HDAC7 (Fig. 1E).

To unambiguously prove the effect on cell proliferation of HDAC7, an inducible version of HDAC7, fused to ER (oestrogen receptor), was re‐introduced in *HDAC7* ^*−/−*^ cells. The same cells expressing the ER alone were also generated. Treatment with 4‐OHT stabilized the expression of the protein (Fig. 1F). Inhibition of nuclear export by leptomycin B treatment proved that, similarly to the endogenous HDAC7, HDAC7‐ER undergoes nuclear/cytoplasmic shuttling (Fig. S1B). The increase in CDKN1A/p21 levels (Fig. 1G,H) and the proliferative defects of *HDAC7* ^*−/−*^ cells (Fig. 1I) were completely rescued by the re‐expression of HDAC7‐ER, but not by the expression of the ER alone (Fig. 1G–I). The rescue of the proliferative deficit was similarly observed after re‐expression of the nuclear resident HDAC7 protein (Fig. S2). In summary, these data demonstrate that HDAC7, when present in the nucleus, sustains MCF10A cell proliferation and represses *CDKN1A* expression. In summary, these data demonstrate that HDAC7 sustains MCF10A cell proliferation, possibly through the control of *CDKN1A*.

### 3.2 HDAC7 and mammosphere formation

**Figure 2:**
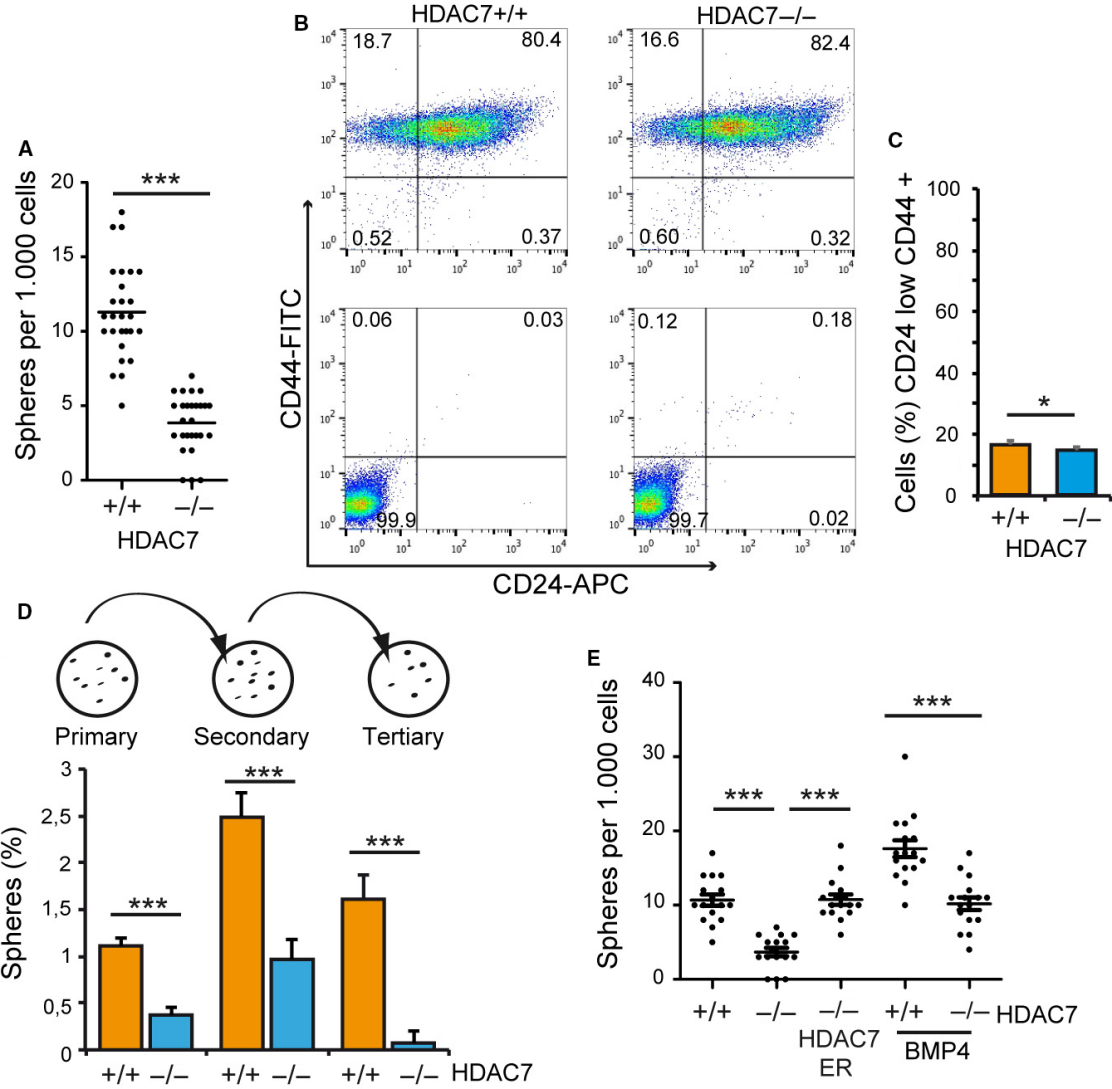
HDAC7 contributes to mammosphere generation in MCF10A cells. (A) Scatter dot plot illustrating the number of mammospheres generated by MCF10A/*HDAC7* ^*+/+*^ or *HDAC7* ^*−/−*^ cells after 10 days in culture. *n* = 9. We marked with ***P(Dunn) < 0.005. (B) Representative FACS analysis of CD44 and CD24 (upper panel) or matched control antibodies (lower panel) in MCF10A/*HDAC7* ^*+/+*^ or *HDAC7* ^*−/−*^ cells. (C) Quantitative analysis of CD44^+^/CD24^low^ cell population as performed by FACS in *HDAC7* ^*+/+*^ or *HDAC7* ^*−/−*^ MCF10A cells. Data are presented as mean ± SD (*n* = 3). We marked with *P(Student) < 0.05. (D) Formation and serial passages of mammospheres as generated by *HDAC7* ^*−/−*^ and *HDAC7* ^*+/+*^ MCF10A cells. Data are presented as mean ± SD (*n* = 3). We marked with ***P(Student) < 0.005. (E) Scatter plots illustrating the number of mammospheres generated by MCF10A *HDAC7* ^*+/+*^ *, HDAC7* ^*−/−*^ *, HDAC7* ^*−/−*^ER and *HDAC7* ^*−/−*^HDAC7‐ER cells, as indicated. For experiments with BMP4, cells were pretreated for 2 days with 15 ng·mL^−1^ of BMP4. *n* = 9. We marked with ***P(Dunn) < 0.005.

MCF10A cells can form spheres with low efficiency when grown in suspension (Amin *et al*., 2016; Clément *et al*., 2017; Qu *et al*., 2015). This *in vitro* assay is commonly used to measure the presence of rare stem‐/progenitor‐like cells (Shaw *et al*., 2012). Since a recent study claimed a role of HDAC7 in breast cancer stem cells (Witt *et al*., 2017), we compared sphere generation by *HDAC7* ^*−/−*^ and *HDAC7* ^*+/+*^ cells. As previously reported (Amin *et al*., 2016; Clément *et al*., 2017; Qu *et al*., 2015), MCF10A can form spheres with low efficiency (Fig. 2A). Of note, sphere formation was almost suppressed in *HDAC7* ^*−/−*^ cells and the rarely generated spheres were reduced in size (Fig. 2A and Fig. S3A,B). The combination of the surface markers CD44^+^/CD24^low^ was used to distinguish stem‐like cells (Amin *et al*., 2016; Clément *et al*., 2017; Shaw *et al*., 2012). We did not observe such a reduction of this population in *HDAC7* ^*−/−*^ cells, to justify the failure in the mammosphere assay (Fig. 2B,C). Serial passages of the mammospheres were performed to assess their self‐renewal capacity. At the third generation, *HDAC7* ^*−/−*^ cells almost exhausted the stem‐like features (Fig. 2D).

The behaviour of stem cells can be influenced by different environmental factors (Shaw *et al*., 2012). Particularly, previous studies have shown that BMP4 can increase the number of mammospheres generated by MCF10A cells (Clément *et al*., 2017). When both *HDAC7* ^*+/+*^ and *HDAC7* ^*−/−*^ cells were treated with BMP4, the number of spheres was augmented. However, fewer spheres were again generated by *HDAC7* ^*−/−*^ cells (Fig. 2E). Importantly, HDAC7 re‐expression fully recovered the defect of KO cells in mammosphere formation (Fig. 2E and Fig. S3A,B).

### 3.3 HDAC7 regulates a repertoire of genes involved in extracellular microenvironment reprogramming

**Figure 3:**
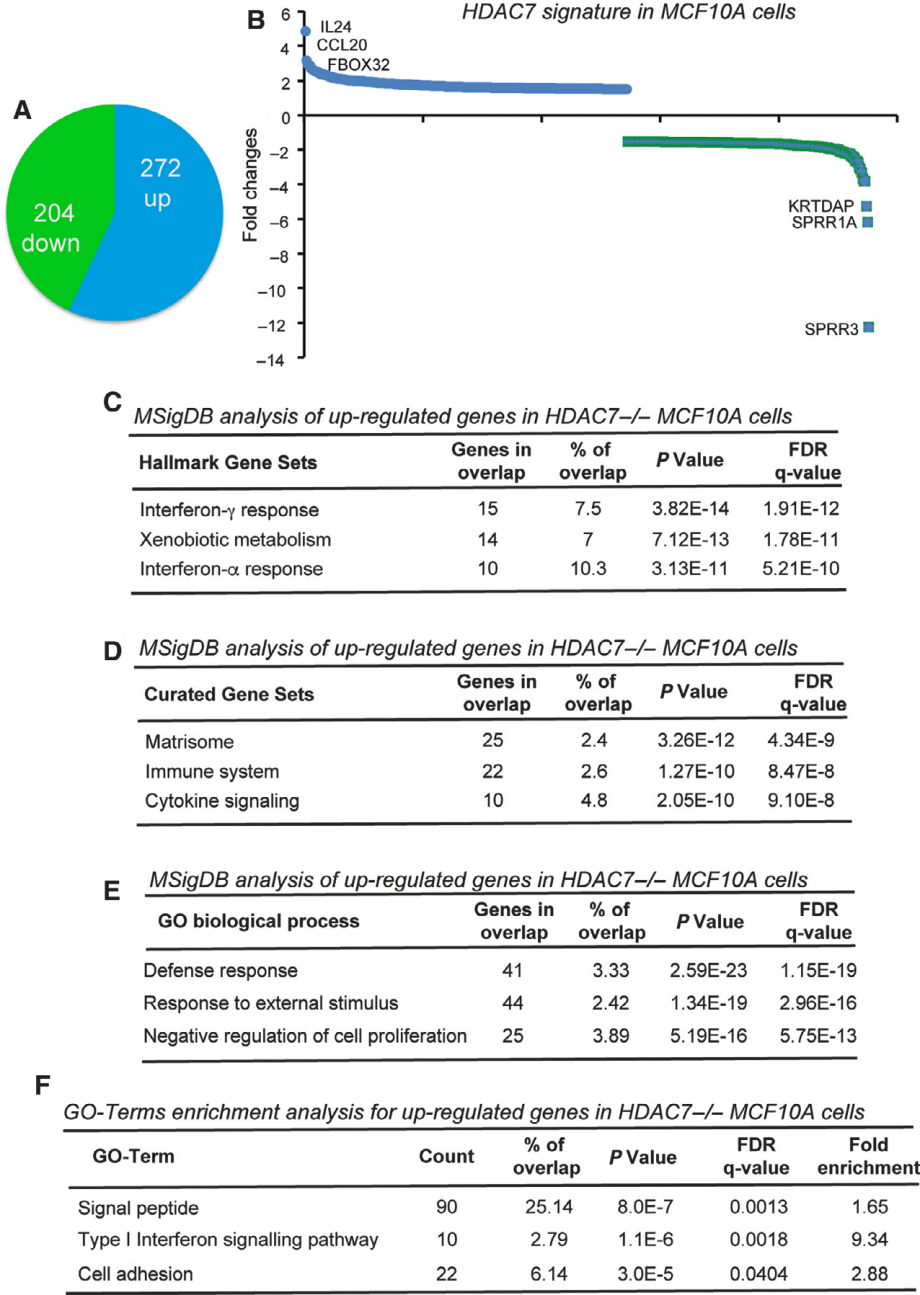
The transcriptome under HDAC7 regulation. (A) Pie‐chart indicating the number of genes significantly up‐ and down‐regulated in HDAC7^*−/−*^ and *HDAC7* ^*−/−*^ER compared to *HDAC7* ^*+/+*^ and *HDAC7* ^*−/−*^HDAC7‐ER MCF10A cells. (B) Histogram representing the HDAC7 signature in MCF10A cells. The top three up‐ and down‐regulated genes are highlighted. (C) GSEA for genes repressed by HDAC7 using MSigDB and the hallmark gene sets. (D) GSEA for genes repressed by HDAC7 using MSigDB and the curated gene sets/canonical pathways. (E) GSEA for genes repressed by HDAC7 using MSigDB and the GO gene sets/biological process. (F) GO term enrichment analysis for genes repressed by HDAC7.

In order to map the genetic programmes supervised by HDAC7, we compared the transcriptomes of *HDAC7* ^*+/+*^ and *HDAC7* ^*−/−*^ cells. To prove that fluctuations in gene expression were a specific consequence of HDAC7 inactivation, we included the gene expression profiles of MCF10A/*HDAC7* ^*−/−*^ */*HDAC7‐ER and its control MCF10A/*HDAC7* ^*−/−*^ */*ER, grown in the presence of 4‐OHT. We found 476 genes (|fc| > 1.5; *P* < 0.05) under HDAC7 influence, of which 272 were up‐regulated and 204 were down‐modulated (Fig. 3A, Table S1). Among these 476 differentially expressed genes, *IL24, CCL20* and *FBXO32* resulted the strongest up‐regulation, whereas *SPRR3, SPRR1A* and *KRTDAP* showed the strongest down‐modulation (Fig. 3B). Since HDAC7 is a transcriptional repressor, we focused the analysis on genes up‐regulated in *HDAC7* ^*−/−*^ cells. The GSEA and the Molecular Signatures Database (MSigDB) analysis showed the up‐regulation of genes involved in the inflammatory response (interferon‐α and γ responses) and the xenobiotic response in *HDAC7* ^*−/−*^ cells (Fig. 3C). ECM, immune system and cytokine signalling resulted in the first three enriched MSigDB curated gene set categories (Fig. 3D). Finally, the top‐ranking GO biological processes were the defence response, the response to external stimulus and the negative regulation of cell proliferation (Fig. 3E). Similarly, the top GO term analysis revealed the enrichment of the GO term cell adhesion, type I interferon signalling pathway and signal peptide in *HDAC7* ^*−/−*^ cells (Fig. 3F). Overall, many genes repressed by HDAC7 modulate the extracellular microenvironment, including the inflammatory response and cell adhesion.

### 3.4 HDAC7 depletion affects a pro‐stemness programme triggered by BMP4

To understand the influence of HDAC7 on the ability of BMP4 to promote mammosphere growth, we compared the gene expression profiles of BMP4‐treated *HDAC7* ^*+/+*^ and *HDAC7* ^*−/−*^ cells. BMP4 promoted the up‐regulation of 182 and 184 genes, respectively, in *HDAC7* ^*+/+*^ and in *HDAC7* ^*−/−*^ cells (|fc > 2.0|; *P* < 0.05) (Fig. S4A and Table S2). Seventy‐five genes were commonly up‐regulated in the two cell lines. As expected, BMP4 triggered the TGF‐β signalling, in both WT and KO cells (Fig. S4B,C). This result implies that the genetic programmes activated by BMP4 are not overtly compromised by the absence of HDAC7, even though some minor changes can be appreciated (Fig. S4B,C). A stronger quantitative difference regards genes down‐regulated by BMP4. One hundred and seventy‐three genes were repressed in the presence of HDAC7 and only 93 in its absence (|fc > 2.0|; *P* < 0.05) (Fig. S4D and Table S3). The epithelial–mesenchymal transition emerged as the genetic programme repressed by BMP4 in both *HDAC7* ^*+/+*^ and *HDAC7* ^*−/−*^ cells (GSEA on chemical and genetic perturbations). When GSEA was done with other gene sets, few overlapping results were found between *HDAC7* ^*+/+*^ and *HDAC7* ^*−/−*^ cells (Fig. S4E,F).

**Figure 4:**
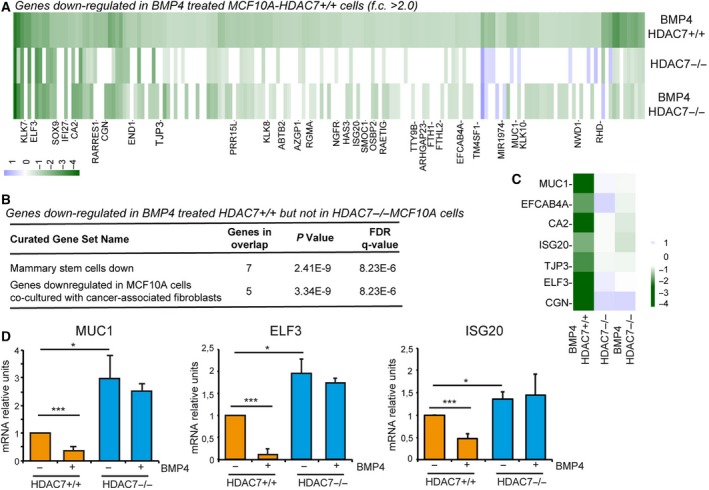
HDAC7 influences the repressive action of BMP4. (A) Genes repressed after BMP4 treatment in MCF10A/*HDAC7* ^*+/+*^ cells were hierarchically clustered with respect to MCF10A/*HDAC7* ^*−/−*^ cells, treated or not with BMP4. The heatmap indicates fold changes. (B) GSEA for genes repressed by HDAC7 using MSigDB and the curated gene sets. (C) Heatmap indicating the mRNA fold changes of the 7 genes belonging to the curated gene set ‘mammary stem cells down’. (D) mRNA expression levels of *MUC1*,* ELF3* and *ISG20* as measured by qRT/PCR in MCF10A/*HDAC7* ^*+/+*^ and MCF10A/*HDAC7* ^*−/−*^ cells treated or not with BMP4. Data are presented as mean ± SD (*n* = 3). We marked with *P(Kruskal–Wallis) < 0.05, ***P < 0.005.

It is reasonable to hypothesize that the reduced pro‐stem activity of BMP4 in *HDAC7* ^*−/−*^ cells (Fig. 2E) could depend on the deficit in repressing few genes important for stemness. These genes should be repressed by BMP4 in WT cells but not in KO cells. Importantly, they should not be repressed in untreated HDAC7^*−/−*^ cells. Hence, we selected all genes repressed by BMP4 in *HDAC7* ^*+/+*^ (fc > 2.0, *n* = 173). Next, their expression was compared with genes repressed after HDAC7 knock‐out and genes repressed by BMP4 treatment in HDAC7^*−/−*^ cells. 31 genes were repressed by BMP4 in a HDAC7‐dependent manner (Fig. 4A). GSEA was performed on this 31‐gene signature and the top curated gene set identified was the ‘mammary stem cells down‐regulated genes’ (Fig. 4B,C). qRT/PCR analysis was performed for *MUC1, ELF3* and *ISG20*. All these three genes were up‐regulated in *HDAC7* ^*−/−*^ cells, down‐regulated in response to BMP4, and the BMP4‐dependent suppression was inefficient in *HDAC7* ^*−/−*^ cells (Fig. 4D).

### 3.5 Identification of a HDAC7‐regulated secretome signature that sustains stemness and predicts a negative prognosis in luminal A breast cancers

**Figure 5:**
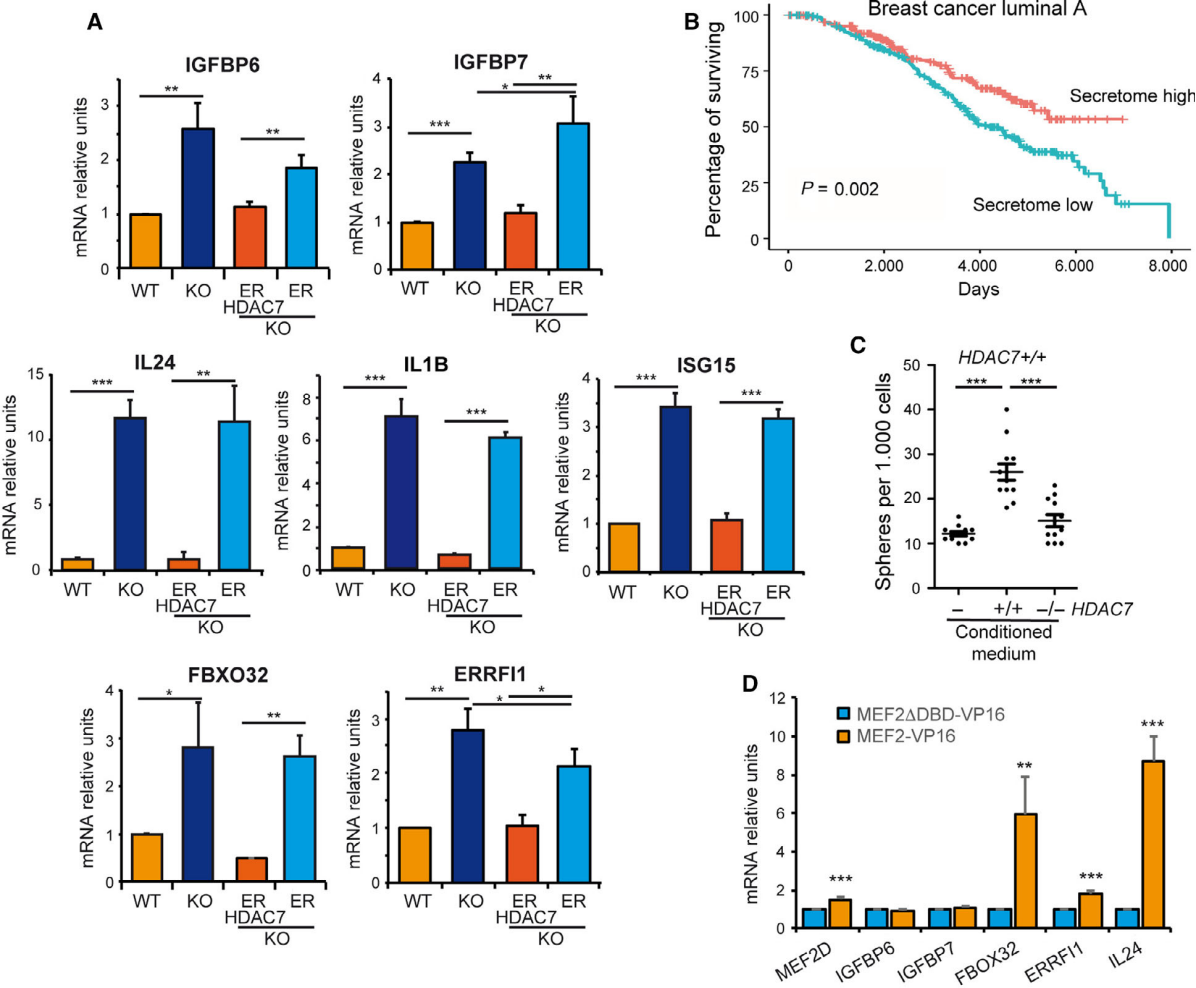
HDAC7 influences the microenvironment. (A) mRNA expression levels of the indicated genes, as measured by qRT/PCR in MCF10A *HDAC7* ^*+/+*^ *, HDAC7* ^*−/−*^ER and *HDAC7* ^*−/−*^HDAC7‐ER cells treated for 36 h with 4‐OHT. Data are presented as mean ± SD (*n* = 3). We marked with *P(Kruskal–Wallis) < 0.05, **P < 0.01, ***P < 0.005. (B) Kaplan–Meier analysis (Wilcoxon test) based on HDAC7 up‐regulated genes coding for 40 secreted factors, using data from 466 luminal A breast cancers. (C) Scatter dot plot illustrating the number of mammospheres generated by *HDAC7* ^*+/+*^ and *HDAC7* ^*−/−*^ MCF10A cells under different environmental conditions. Conditioned medium from 2D cultures of *HDAC7* ^*+/+*^ cells was generated and used as indicated in material and methods. *n* = 12. We marked with ***P(Dunn) < 0.005. (D) mRNA expression levels of selected HDAC7 target genes in MCF10A cells expressing MEF2‐VP16‐ER or the mutant MEF2ΔDBD‐VP16‐ER as measured by qRT/PCR analysis, following 36 h of 4‐OHT treatment in 2D culture. Data are presented as mean ± SD (*n* = 3). We marked with **P(Student) < 0.01, ***P < 0.005.

The transcriptomic analysis revealed that HDAC7 represses the expression of 40 genes encoding for secreted factors that we named ‘HDAC7‐secretome signature’ (Table S4). These include: (a) chemokine ligands *CCL5, CCL20, CCL24*, (b) *ISG15*, which acts also extracellularly to favour IFN‐γ secretion (Zhang *et al*., 2015), (c) interleukins *IL1B* and *IL24*, and (d) elements of the insulin‐like growth factor (IGF) signalling (*IGFBP6, IGFB7, IGFL2*). The new HDAC7 targets such as the inhibitors of IGF signalling (*IGFBP6* and *IGFBP7)*, interleukins *IL1B* and *IL24* and the interferon‐inducible gene *ISG15* were validated by qRT/PCR (Fig. 5A). We also included *ERRFI1,* which encodes for a negative regulator of the RTK signalling (Segatto *et al*., 2011), and the E3 ligase *FBXO32/Mafbx/Atrogin1*, involved in muscular atrophy (Bodine and Baehr, 2014). All tested genes were up‐regulated in *HDAC7* ^*−/−*^ cells and re‐repressed upon HDAC7 re‐activation (Fig. 5A). GSEA proved that a role of HDAC7 in the regulation of the secretome is not restricted to MCF10A cells. Part of this signature is up‐regulated also in human endothelial progenitor cells, after HDAC7 silencing. Importantly, also in these cells *IL24* is the most responsive gene (Fig. S5A,B; Wei *et al*., 2018).

The influence of HDAC7 on the microenvironment could have important implications *in vivo*. When luminal A breast cancer patients were stratified according to the expression levels of the HDAC7‐secretome signature (Curtis *et al*., 2012), patients with high levels of the signature showed a better prognosis (Fig. 5B). This effect could be due to an impairment on cancer stem properties caused by the activation of this signature. To prove this hypothesis, we compared the conditioned medium taken from *HDAC7* ^*+/+*^ and *HDAC7* ^*−/−*^ cells for the capability of sustaining mammosphere generation. Conditioned medium from *HDAC7* ^*+/+*^ cells strongly increased the number of spheres generated, whereas the medium from *HDAC7* ^*−/−*^ cells did not provide advantages (Fig. 5C).

HDAC7 indirectly controls gene expression by forming complexes with TF. Important partners of HDAC7 are the MEF2 family members (Di Giorgio *et al*., 2018). To understand whether genes under HDAC7 influence are MEF2 targets, we expressed an inducible hyperactive version of MEF2 in MCF10A cells (Clocchiatti *et al*., 2015). As a control, a MEF2 deleted in the DNA binding domain was used. qRT/PCR analysis indicated that *FBXO32*,* ERRFI1* and *IL24* are under MEF2 regulation. By contrast, *IGFBP6* and *IGFBP7* are not MEF2 targets (Fig. 5D).

### 3.6 Mapping HDAC7 activities at the genomic level

The above study indicates that HDAC7 can influence the expression of genes also independently from MEF2 TFs. These genes could be direct or indirect targets of HDAC7. As a first step to clarify the genomic functions of HDAC7, we performed chromatin immunoprecipitation sequencing (ChIP‐seq) in *HDAC7* ^*+/+*^ and *HDAC7* ^*−/−*^ cells. We also compared the distribution of the histone H3 acetylated on lysine 27 (H3K27ac), a marker of transcriptionally active promoters and enhancers.

**Figure 6:**
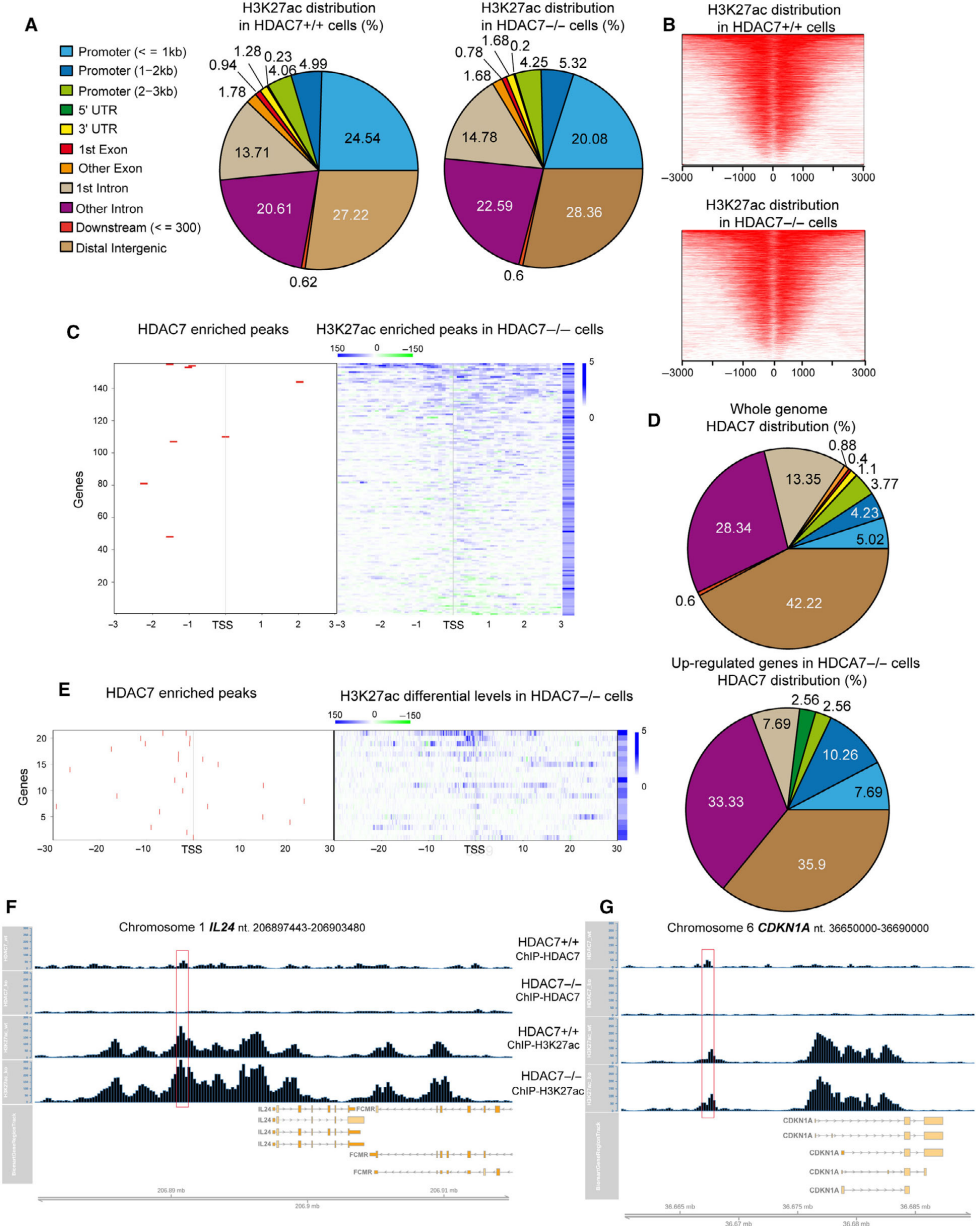
Loss of HDAC7 leads to local increases in H3K27ac. (A) Genomic distribution of H3K27ac‐enriched peaks in *HDAC7* ^*+/+*^ and *HDAC7* ^*−/−*^ MCF10A cells, as identified by homer. (B) ChIP‐seq density heatmaps of H3K27ac‐enriched peaks in *HDAC7* ^*+/+*^ and *HDAC7* ^*−/−*^ MCF10A cells in a region of ±3 kb around the TSS of the closest genes. (C) Heatmaps showing the differences in H3K27ac enrichment between *HDAC7* ^*−/−*^ and *HDAC7* ^*+/+*^ MCF10A cells (right panel) and the associated presence of HDAC7‐enriched peaks in *HDAC7* ^*+/+*^ MCF10A cells (left panel) in a region of ±3 kb around the TSS of the 155 microarray‐defined HDAC7 repressed genes. The small heatmap on the right shows the mRNA expression fold changes. (D) Genomic distribution of all the HDAC7‐enriched peaks (top panel), or only those associated with genes up‐regulated in microarray (bottom panel), in *HDAC7* ^*+/+*^ MCF10A, as defined by homer. (E) Heatmaps showing HDAC7‐enriched peaks (left panel) and the associated differences in H3K27ac enrichment between *HDAC7* ^*−/−*^ and *HDAC7* ^*+/+*^ MCF10A cells (right panel) in a region of ±30 kb around the TSS of a subset of 21 microarray‐defined HDAC7 repressed genes. (F) Detailed view of the H3K27ac and HDAC7 tracks at the *IL24* locus in *HDAC7* ^*−/−*^ and *HDAC7* ^*+/+*^ MCF10A cells. Gene structure and chromosomal location are shown, with the red box highlighting the enriched peaks. (G) Detailed view of H3K27ac and HDAC7 tracks at the *CDKN1A* locus in *HDAC7* ^*−/−*^ and *HDAC7* ^*+/+*^ MCF10A cells. Gene structure and chromosomal location are shown, with the red box highlighting the enriched peaks.

Overall, H3K27ac‐enriched regions were increased in the absence of HDAC7 (139.328 peaks compared to 95.419 in *HDAC7* ^*+/+*^ cells). The analysis of the genomic distributions of these marks revealed an increase in H3K27ac at introns and distal intergenic regions in *HDAC7* ^*−/−*^ cells (Fig. 6A). We found 5072 enriched peaks for HDAC7 in WT cells. Nine hundred and eighty‐nine and 1380 of them colocalized (±1 kb interval) with H3K27ac peaks, respectively, in *HDAC7* ^*+/+*^ and *HDAC7* ^*−/−*^ cells. This evidence suggests that de novo H3K27ac can be elicited after HDAC7 suppression.

Comparison of the H3K27ac peak distribution with respect to the TSS in *HDAC7* ^*+/+*^ and *HDAC7* ^*−/−*^ cells confirmed that HDAC7 does not play a global activity, being the pattern of H3K27ac distribution almost superimposable between the two cell lines (Fig. 6B and Fig. S6). Despite this, HDAC7 could operate on selected TSS. To verify this hypothesis, we focused the analysis on the H3K27ac status at the TSS of the 272 genes up‐regulated in *HDAC7* ^*−/−*^ cells. We excluded transcripts with undefined functional annotations, thus resulting in 155 genes.

In *HDAC7* ^*−/−*^ cells, the H3K27ac status at the ∓3 kb regions around the TSSs evidenced a robust increase for the vast majority of the 155 genes. The H3K27ac increase was more frequently observed downstream from the TSS (Fig. 6C). *IL24*,* OPN3* and *IRF6* were the top genes for H3K27ac rise, within the ∓3 kb region. The regions where these three genes displayed a strong increase of H3K27ac (around ∓1 kb from the TSS), were, at the same time, also enriched for HDAC7 binding peaks (*IL24* −1580; *OPN3* −825; *IRF6* −962 from the TSS). Only 8 loci, among the 155 analysed, showed enriched peaks for HDAC7 in regions close to the TSS. It is possible that the deacetylase binds other regulative elements located more distally, with respect to the TSS (Azagra *et al*., 2016). Indeed, the genomic distribution of HDAC7 peaks evinced that approx. 70% of HDAC7 binding occurred in intergenic and intronic regions, while only a reduced percentage (approximately 26%, including the first intron) in regions near to the TSS and only 13% at promoters (Fig. 6D). Out of the 2118 HDAC7 peaks located in the intergenic regions, 204 were acetylated in WT cells (±1 kb) and 272 in KO cells. In summary, the vast majority of intergenic regions bound by HDAC7 seem to be transcriptionally inactive and only a fraction (approx. 9.6%) are in open chromatin. Removal of HDAC7 increases these H3K27ac intergenic regions up to 12.8%. It is possible that within these regions, some distal regulative elements, such as enhancers, could be found.

When the same analysis was performed on the 155 genes up‐regulated in *HDAC7* ^*−/−*^ cells, the percentage of promoter sequences enriched for HDAC7 binding raised up to more than 20%. Based on these evidences, we analysed H3K27ac and HDAC7 peak distributions in a wider region (−30 kb/30 kb) from the TSS of the 155 genes. In this interval, 21 out of 155 loci showed at least an enriched peak for HDAC7 (Fig. 6E, Table S5). In some cases, HDAC7 peaks colocalized with regions where H3K27ac was increased in a HDAC7‐dependent manner. In other regions, this colocalization was not observed. In 50% of cases, at least one putative MEF2 binding site was present within HDAC7‐enriched peaks associated with these 21 genes (*P*‐value ≤ 0.05).

As representative examples of the ChIP‐seq data, the *IL24* and *CDKN1A* loci are shown in Fig. 6F,G. These genes emerged as two HDAC7 direct targets, which show the colocalization of HDAC7 and H3K27ac peaks.

### 3.7 Roles of HDAC7 in the transformation of human breast epithelial cells

**Figure 7:**
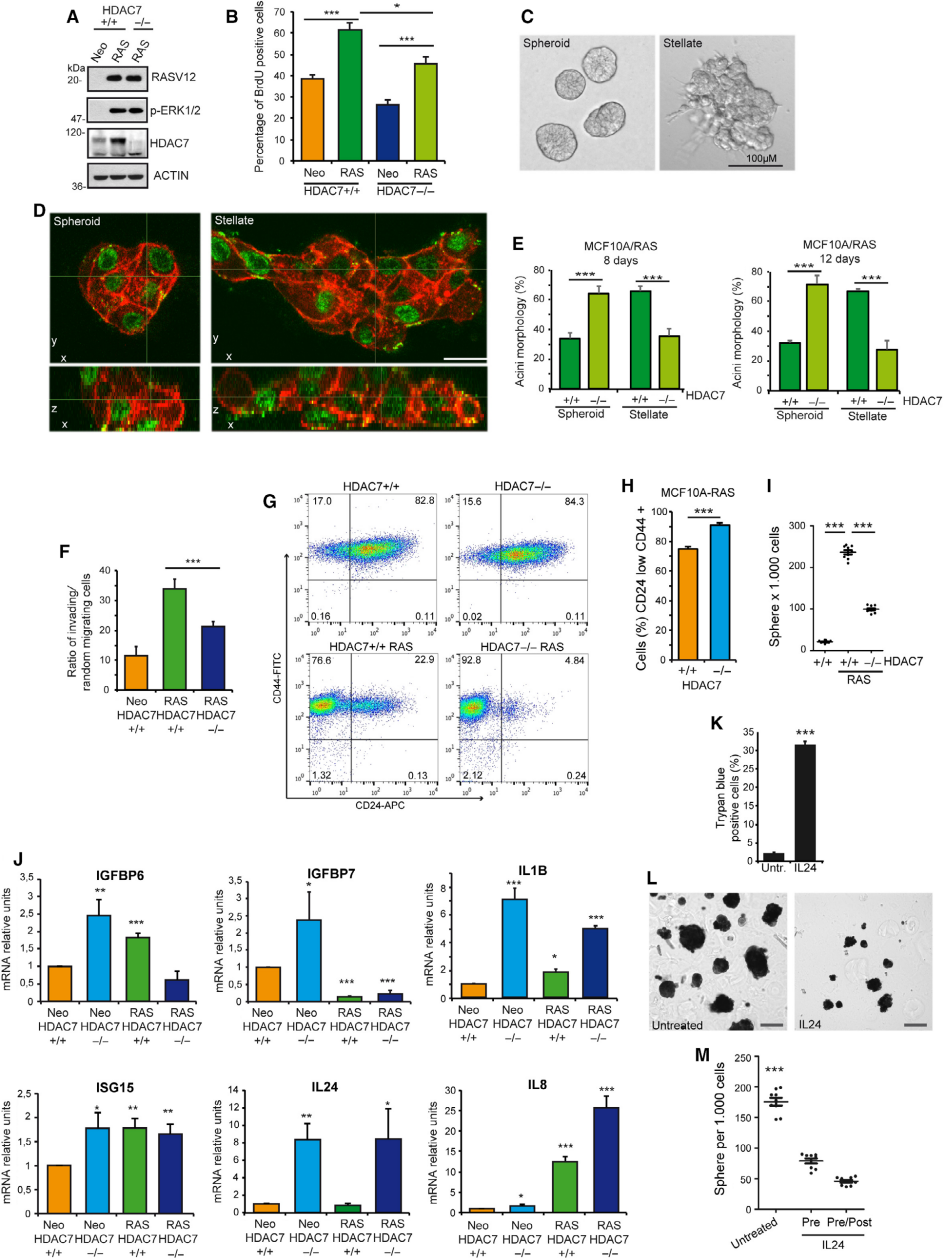
HDAC7 influences the transformation properties of RAS‐transformed cells. (A) Immunoblot analysis of MCF10/RAS‐transformed cells expressing or not HDAC7. Cellular lysates were generated and after blotting incubated with the indicated antibodies. Actin was used as loading control. (B) S‐phase determination by BrdU incorporation in RAS‐transformed MCF10A/*HDAC7* ^*+/+*^ and MCF10A/*HDAC7* ^*−/−*^ cells. Data are presented as mean ± SD (*n* = 3). We marked with *P(Kruskal–Wallis) < 0.05, ***P < 0.005. (C) Representative phase‐contrast microscope images of typical spheroid and stellate‐shaped acini obtained after 8 days of 3D culture. (D) Representative confocal images showing the comparison between typical stellate and spheroid acini generated by MCF10A/RAS cells grown under 3D conditions. AF546‐phalloidin was used to stain F‐actin (red), and nuclei were stained with anti‐HMGA2 (green). In the lower part, the Z‐section is shown as indicated. Bar 50 μm. (E) Percentage of spheroid and stellate acini in MCF10A/RAS *HDAC7* ^*+/+*^ or *HDAC7* ^*−/−*^, as indicated. Acini were scored after 8 and 12 days in culture. Data are presented as mean ± SD (*n* = 3). We marked with ***P(Student) < 0.005. (F) Transwell migration assay of MCF10A/RAS *HDAC7* ^*+/+*^ or *HDAC7* ^*−/−*^. The percentage of migrating cells was calculated as the ratio between the number of invasive cells and the random migrating cells. Data are presented as mean ± SD (*n* = 3). We marked with ***P(Student) < 0.005. (G) Flow cytometry analysis of CD44 and CD24 markers in MCF10A/*HDAC7* ^*+/+*^ or *HDAC7* ^*−/−*^ cells (upper panel) compared to RAS‐transformed cells (lower panel). (H) Quantitative analysis of CD44^+^/CD24^low^ cell population as performed by FACS. Data are from three independent experiments, ±SD. We marked with ***P(Student) < 0.005. (I) Scatter dot plot illustrating the number of mammospheres generated by MCF10A/*HDAC7* ^*+/+*^ *,* MCF10A/RAS *HDAC7* ^*+/+*^ or *HDAC7* ^*−/−*^ cells, grown in a semisolid medium containing 0.5% methylcellulose. *n* = 9. We marked with ***P(Dunn) < 0.005. (J) mRNA expression levels of the HDAC7‐repressed genes coding for microenvironmental factors, as measured by qRT/PCR in the indicated cell lines. Data are presented as mean ± SD (*n* = 3). We marked with *P(Kruskal–Wallis) < 0.05, **P < 0.01, ***P < 0.005. (K) Cell death quantification after incubation of MCF10A/RAS cells for 48 h with 20 ng·mL^−1^ of IL24. Data are presented as mean ± SD (*n* = 3). We marked with ***P(Student) < 0.005. (L) Representative images of mammospheres generated by MCF10A/RAS pretreated with 20 ng·mL^−1^ of IL24 and grown in the presence of 20 ng·mL^−1^ of IL24 in the MM and the untreated control after 10 days in culture. Mammospheres were stained with MTT. Scale bar, 100 μm. (M) Scatter dot plot illustrating the number of mammospheres generated by MCF10A/RAS cells grown for 48 h in the presence of 20 ng·mL^−1^ of IL24 before seeding in MM (Pre), growth in control MM (untreated) or pretreated with IL24 (20 ng·mL^−1^) in MM and next grown for 10 days in MM with IL24 (20 ng·mL^−1^) (Pre/Post). *n* = 9, ***P(Dunn) < 0.005.

To explore the contribution of HDAC7 to breast cell transformation and to CSC features, we introduced the RAS oncogene in *HDAC7* ^*−/−*^ and in *HDAC7* ^*+/+*^ cells. RAS elicits a transformed phenotype, characterized by invasive and anchorage‐independent growth (Liu *et al*., 2013; Moon *et al*., 2000). RAS was similarly expressed in the two cell lines, and ERK phosphorylation was dramatically augmented, independently from HDAC7 (Fig. 7A). Interestingly, HDAC7 levels were increased in cells overexpressing RAS (Fig. 7A). Comparative analysis of HDAC7 subcellular localization revealed that in MCF10A cells, the deacetylase shows a uniform nuclear/cytoplasmic localization. In RAS‐transformed cells, HDAC7 localization is more heterogeneous. Some cells show higher nuclear staining while others a stronger cytosolic signal (Fig. S7).

Cell proliferation was augmented by RAS in both *HDAC7* ^*−/−*^ and *HDAC7* ^*+/+*^ cells. However, in the absence of HDAC7, RAS‐transformed cells retain the S‐phase deficit previously described (Figs 1B and 7B). When grown in 3D conditions, RAS‐transformed cells adopt an invasive stellate growth pattern (Giunciuglio *et al*., 1995). We observed that in 3D conditions, MCF10A/RAS cells can generate acini with a normal spheroid morphology, as well as stellate acini, which show features of invasive growth (Fig. 7C,D). Elimination of HDAC7 reduces the invasive growth in 3D culture of MCF10A/RAS cells (Fig. 7E). To confirm the role of HDAC7 in sustaining an invasive behaviour, MCF10A/RAS, *HDAC7* ^*+/+*^ or *HDAC7* ^*−/−*^ cells were plated on Matrigel‐coated transwell filters to evaluate their migratory properties. As expected, RAS enhanced invasion in Matrigel, but, in the absence of HDAC7, this invasive phenotype was reduced (Fig. 7F).

When RAS‐transformed cells were analysed for the surface markers CD44 and CD24, the cytofluorimetric analysis indicated a dramatic increase in the stem‐like population (Fig. 7G,H). The increased stem features of MCF10/RAS cells were confirmed by the compelling increase in the numbers of mammospheres generated, compared to untransformed cells (Fig. 7I). Importantly, the absence of HDAC7 dramatically reduced the mammosphere generation also in MCF10/RAS cells (Fig. 7I). This reduction, in conjunction with the absence of effects on the CSC population, as determined by CD44^+^/CD24^low^ markers (Fig. 7H), suggests that the impact of HDAC7 on the microenvironment could be again a key aspect to sustain the growth of CSCs.

This consideration prompted us to evaluate whether genes, encoding for secreted factors and up‐regulated in HDAC7 KO cells, were regulated also in MCF10A/RAS cells. Secreted factors, specifically up‐regulated in *HDAC7* ^*−/−*^ cells also in the presence of RAS, are strong candidates as suppressors of mammosphere growth. *ISG15, IGFBP6, IGFBP7* and *IL24* expression levels were evaluated (Fig. 7J). *IL8/CXCL8,* a well‐known RAS target that enhances cell proliferation, was included as control (Sparmann and Bar‐Sagi, 2004; Zheng *et al*., 2015). *IGFBP7* was strongly repressed by RAS. *IGFBP6*, instead, was up‐regulated by RAS through a HDAC7‐dependent mechanism. *ISG15* was modestly up‐regulated by RAS. As expected, *IL8* was strongly up‐regulated by RAS and this up‐regulation was even stronger in the absence of HDAC7. Finally, *IL1B* and *IL24* were strongly up‐regulated in a HDAC7‐dependent and RAS‐independent manner. In summary, these results indicate that part of the HDAC7‐regulated secretome can be influenced during cellular transformation, also independently from HDAC7. The deacetylase maintains a strong repressive influence versus *IL24, IL1B* and *IL8*.

Since *IL24* is an important HDAC7 target and since its antiproliferative activities are well documented, its repression could be critical to favour a proficient microenvironment for stem‐like cells growth. To verify this hypothesis, we treated MCF10A/RAS cells with IL24. We confirmed previous studies showing that IL24 can trigger a certain amount of apoptosis in cancer cells (Fig. 7K). To perform the mammosphere assay, only trypan blue‐negative healthy cells were used. Mammosphere generation by MCF10A/RAS cells was dramatically impaired by IL24 (Fig. 7L). The quantitative analysis proved the inhibitory effect of IL24 on mammosphere generation and growth, when used in the pretreatment, as well as when its presence was maintained throughout the assay (Fig. 7M).

## 4 Discussion

The contribution of class IIa HDACs to cell proliferation and transformation is underestimated. There are some evidences about the transforming potential of HDAC7 and its activity as oncogene (Di Giorgio *et al*., 2013; Lei *et al*., 2017; Paluvai *et al*., 2018; Peixoto *et al*., 2016; Rad *et al*., 2010). Here, we have proved that, in human mammary epithelial cells, HDAC7 influences multiple aspects of the transformation process including proliferation, invasion and stemness. The transcriptomic analysis revealed that a consistent pool of genes repressed by HDAC7 encodes for secreted and plasma membrane proteins. In particular, the expression of inflammatory and antiproliferative cytokines, mediators of the immune response and negative regulators of IGF signalling were repressed by HDAC7. This specific milieu of factors is particularly critical to sustain the growth of stem‐like cells. Importantly, repression of this microenvironmental signature correlates with the aggressiveness of luminal A breast cancers. Not surprisingly, since HDAC7 is regulated at multiple levels, including protein stability and nuclear–cytoplasmic shuttling (Di Giorgio and Brancolini, 2016), we have not found a correlation between its mRNA levels and luminal A tumour aggressiveness.

A role of HDAC7 in the regulation of the microenvironment was previously suggested (Peixoto *et al*., 2016). Here, we have proved that the microenvironment is influenced by HDAC7 and it is involved in the sustainment of the stem‐like population. Among microenvironmental genes influenced by HDAC7, we found *IGFBP6* and *IGFBP7*, proteins that can buffer IGF signalling (Bach, 2015; Evdokimova *et al*., 2012). IGFBP7 expression was strongly repressed also by RAS, regardless of HDAC7. Hence, IGFBP7 could represent a common target of different signalling pathways to sustain stem cell features. In this respect, a role of IGFBP7 as inhibitor of the expansion and aggressiveness of tumour stem‐like cells was recently proved *in vivo* (Cao *et al*., 2017).

HDAC7 represses also some chemokines and cytokines. In the case of *IL24*, the involvement of MEF2s is plausible. Abrogation of IL24 repression, following HDAC7 deletion, seems to be a critical antiproliferative signal. In fact, addition of this cytokine dramatically inhibited mammosphere generation by MCF10A/RAS cells.

*IL24/MDA‐7*, originally identified as a gene whose expression is induced during melanoma differentiation, inhibits tumour growth in different contexts (Bhutia *et al*., 2013; Menezes *et al*., 2014). IL24 belongs to the IL10 gene family and exerts different antitumoural activities such as induction of apoptosis, suppression of invasion/metastasis and anti‐angiogenic effects (Menezes *et al*., 2014). Different mechanisms have been proposed to explain its pro‐apoptotic activity including intracellular and environmental signalling (Bhutia *et al*., 2013; Dash *et al*., 2014; Lebedeva *et al*., 2002; Menezes *et al*., 2014).

HDAC7 binds the promoter of IL24 at −1580 and −6574 from the TSS and influences the H3K27 acetylation status in a wider genomic region. Genomic analysis has defined that HDAC7 more frequently binds intergenic regions or regions distal from TSS. Only in a small percentage of cases, the binding of HDAC7 was observed close to the TSS. Not surprisingly for a repressor, HDAC7 can be found in regions marked by low H3K27ac levels, which should indicate the maturation of a repressed chromatin status. However, HDAC7 was detected also in transcriptionally active domains, characterized by high amounts of H3K27ac. Here, it actively contributes to buffer the acetylation levels.

The impact of HDAC7 on cytokine production is not limited to IL24. *IL1B* was identified among genes up‐regulated in *HDAC7* ^*−/−*^ cells, and *IL8* was strongly up‐regulated in RAS‐transformed *HDAC7* ^*−/−*^ cells. We observed that also *IL32* is repressed by HDAC7 (data not show). Since these cytokines can exert opposite effects on proliferation and stemness and operate also as paracrine factors, further studies will be necessary to clarify the influence of HDAC7 on the microenvironment.

In addition to the microenvironment, also cellular factors repressed by HDAC7 contribute to growth/survival of stem‐like cells. In fact, addition of BMP4 was not sufficient to fully recover the defect of *HDAC7* ^*−/−*^ cells. Among genes, possibly involved in this response, the panel of 7 genes repressed by BMP4 in a HDAC7‐dependent manner and consistently down‐regulated in mammary stem cells (Lim *et al*., 2010) are good candidates that should be tested in the future.

## 5 Conclusions

In conclusion, our study adds another piece of evidence about the contributions of HDAC7 to multiple proliferative options, including the regulation of stem‐like cells. Overall, our results encourage further efforts to discover and evaluate HDAC7‐specific inhibitors (Di Giorgio *et al*., 2015) in a therapeutic perspective.

## Conflict of interest

The authors declare no conflict of interest.

## Author contributions

CB designed research; RP, ED, EDG and CB analysed data; VC, EDG and MM performed research; and VC, EDG, ED, CB wrote the paper.

## Supporting information


**Fig. S1.** Characterization of MCF10A HDAC7^*−/−*^ cells.

**Fig. S2.** The nuclear resident version of HDAC7 rescues the proliferative defect of HDAC7^*−/−*^ cells.

**Fig. S3.** HDAC7 and mammospheres generation potential.

**Fig. S4.** The transcriptomic response to BMP4 treatment.

**Fig. S5.** HDAC7 and the secretome.

**Fig. S6.** Distance from TSS of H3K27ac peaks in *HDAC7* ^*+/+*^ and *HDAC7* ^*−/−*^ MCF10A cells.

**Fig. S7.** Subcellular localization of HDAC7 in MCF10A transformed or not with RAS.

**Table S1.** List of the 476 genes regulated by HDAC7.

**Table S2.** List of the genes regulated by BMP4 in *HDAC7* ^*+/+*^ cells.

**Table S3.** List of the genes regulated by BMP4 in *HDAC7* ^*−/−*^ cells.

**Table S4.** List of the 40 genes characterizing the signature of HDAC7‐repressed secreted factors.

**Table S5.** HDAC7wt enriched peaks associated with microarray up‐regulated genes.
