# Supplementary figures and images for: HDAC7‐mediated control of tumour microenvironment maintains proliferative and stemness competence of human mammary epithelial cells
Source: Mol Oncol. 2019 Jun 27;13(8):1651–68. doi: 10.1002/1878-0261.12503 (PMC6670296; doi:10.1002/1878-0261.12503)

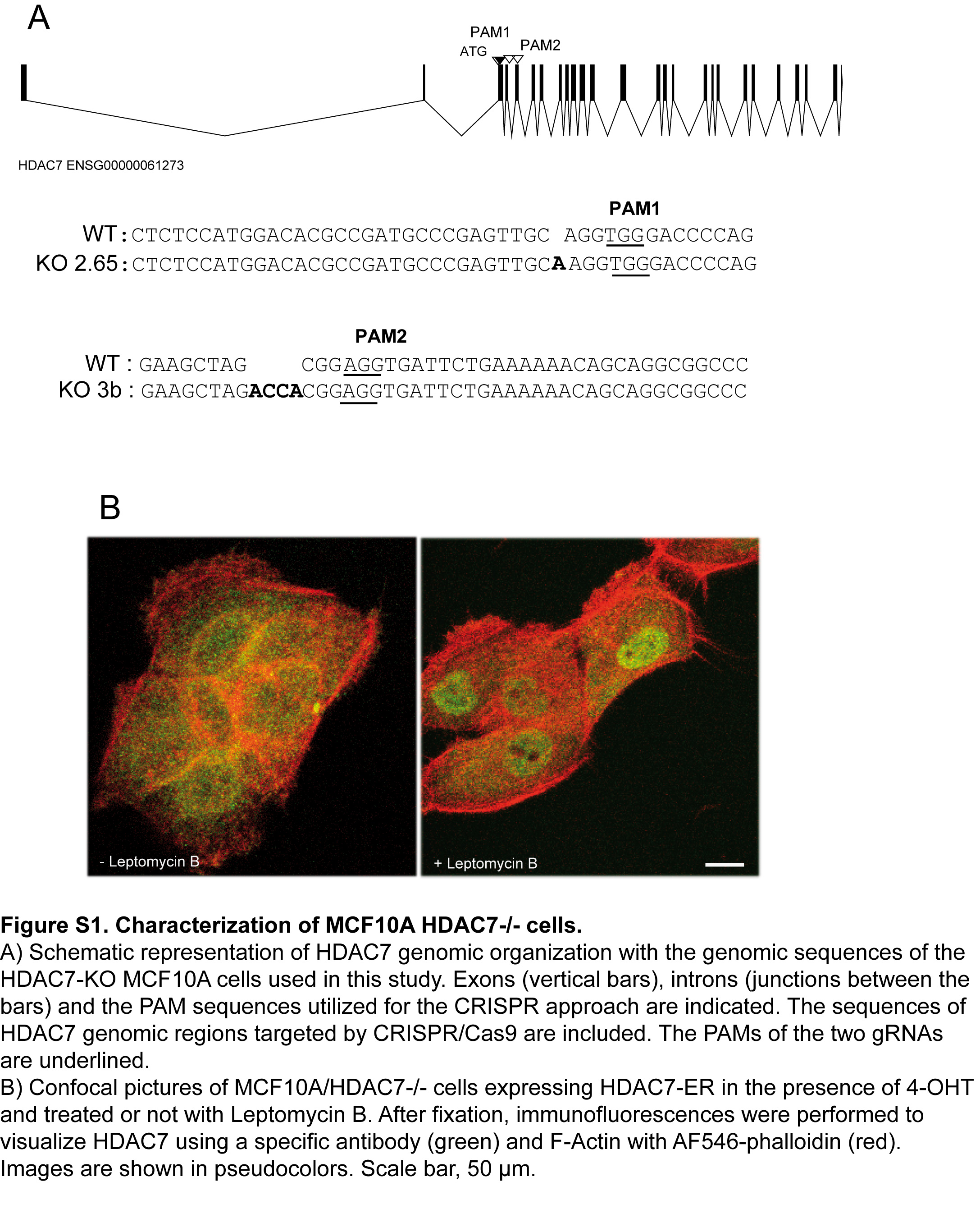

Supplement: Supplementary file 1 — Fig. S1. Characterization of MCF10A HDAC7−/− cells. [file MOL2-13-1651-s001.jpg]

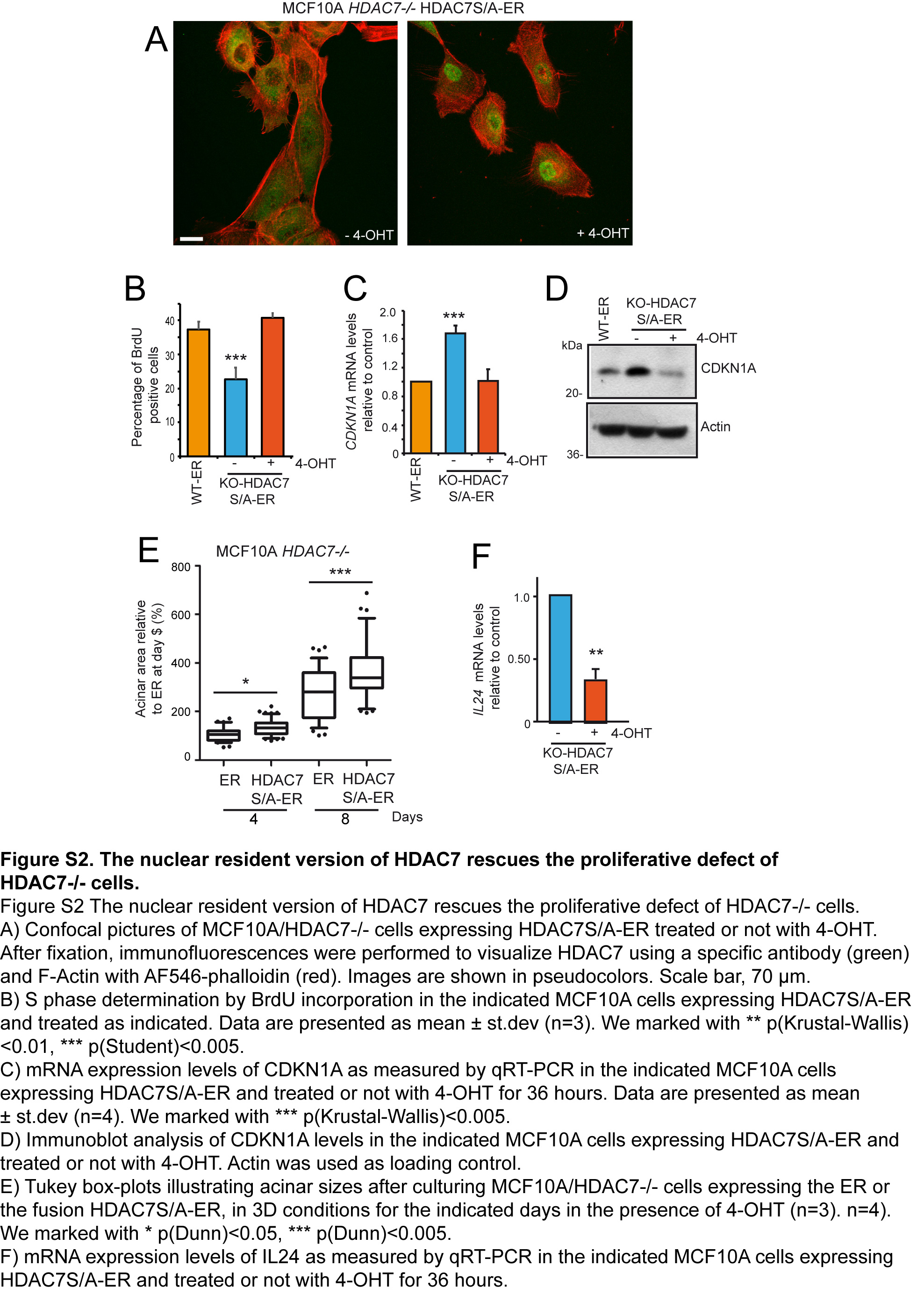

Supplement: Supplementary file 2 — Fig. S2. The nuclear resident version of HDAC7 rescues the proliferative defect of HDAC7−/− cells. [file MOL2-13-1651-s002.jpg]

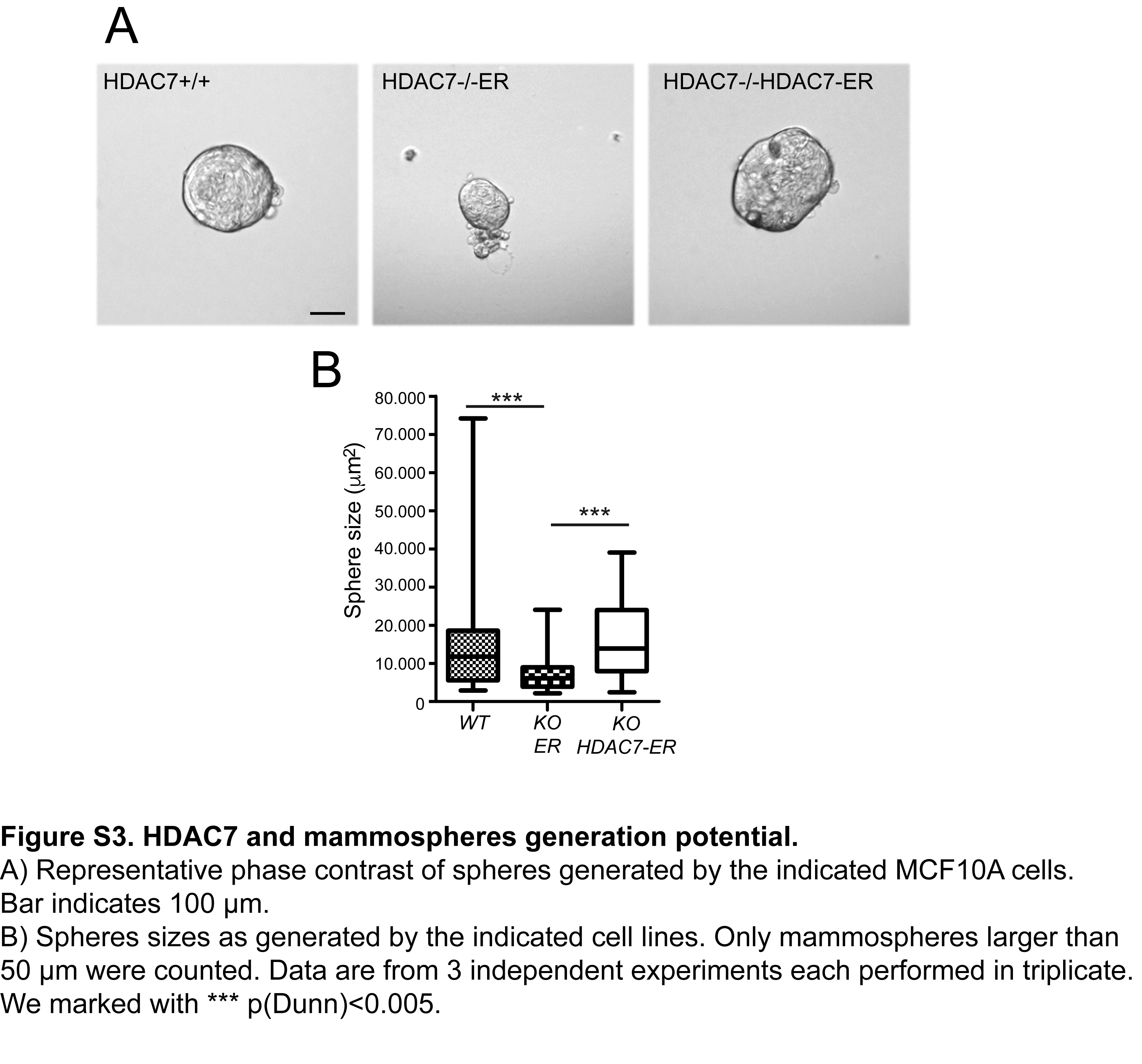

Supplement: Supplementary file 3 — Fig. S3. HDAC7 and mammospheres generation potential. [file MOL2-13-1651-s003.jpg]

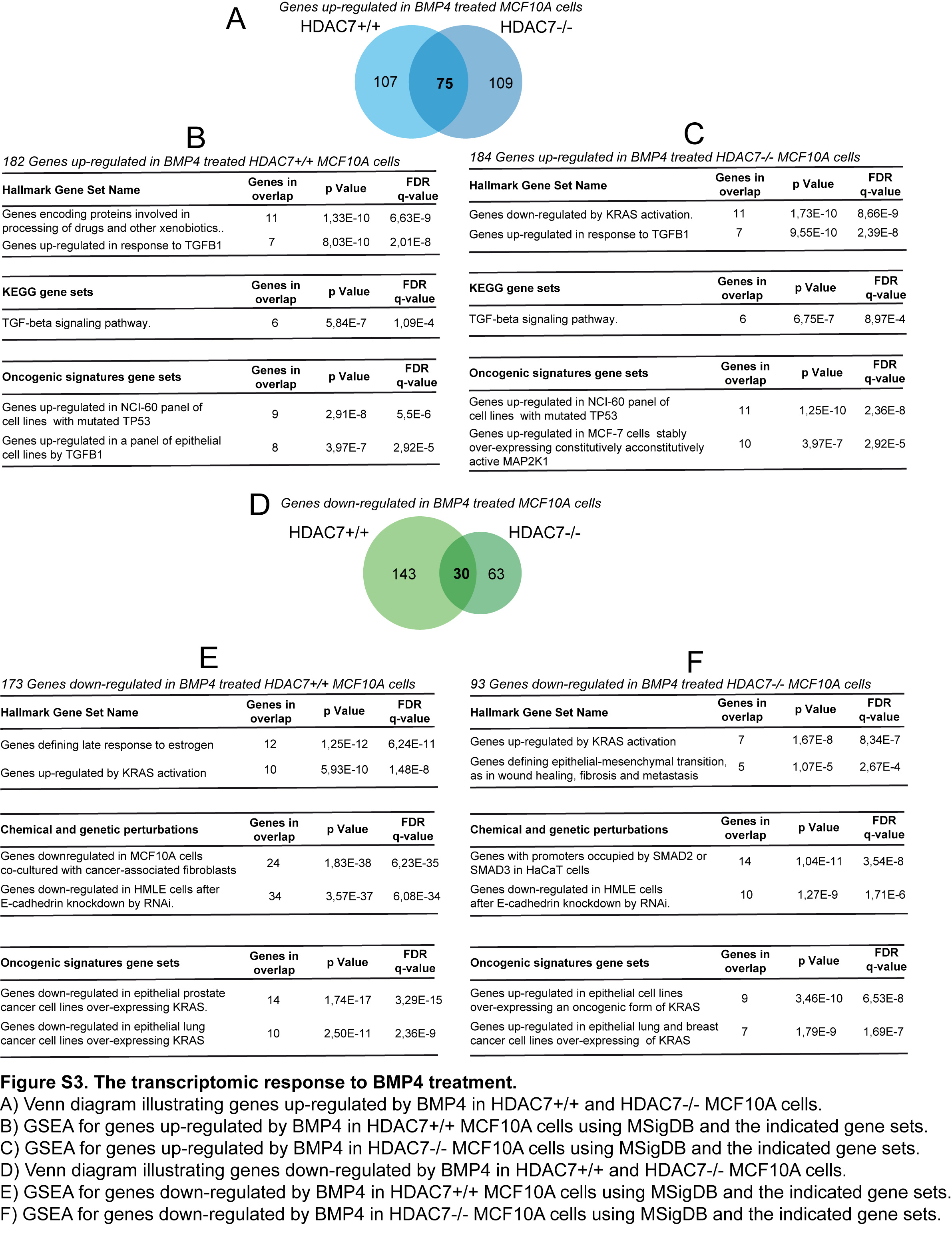

Supplement: Supplementary file 4 — Fig. S4. The transcriptomic response to BMP4 treatment. [file MOL2-13-1651-s004.jpg]

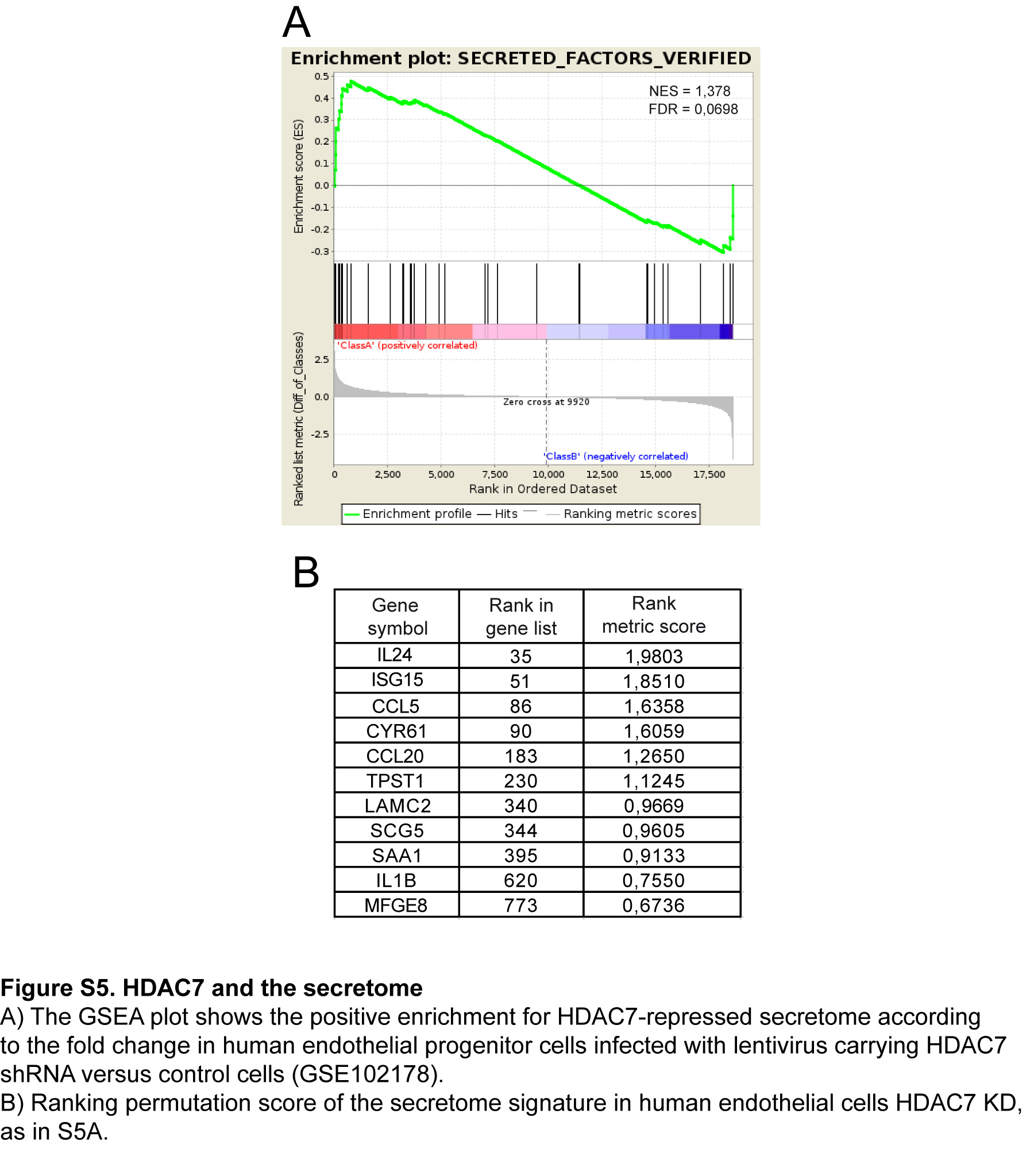

Supplement: Supplementary file 5 — Fig. S5. HDAC7 and the secretome. [file MOL2-13-1651-s005.jpg]

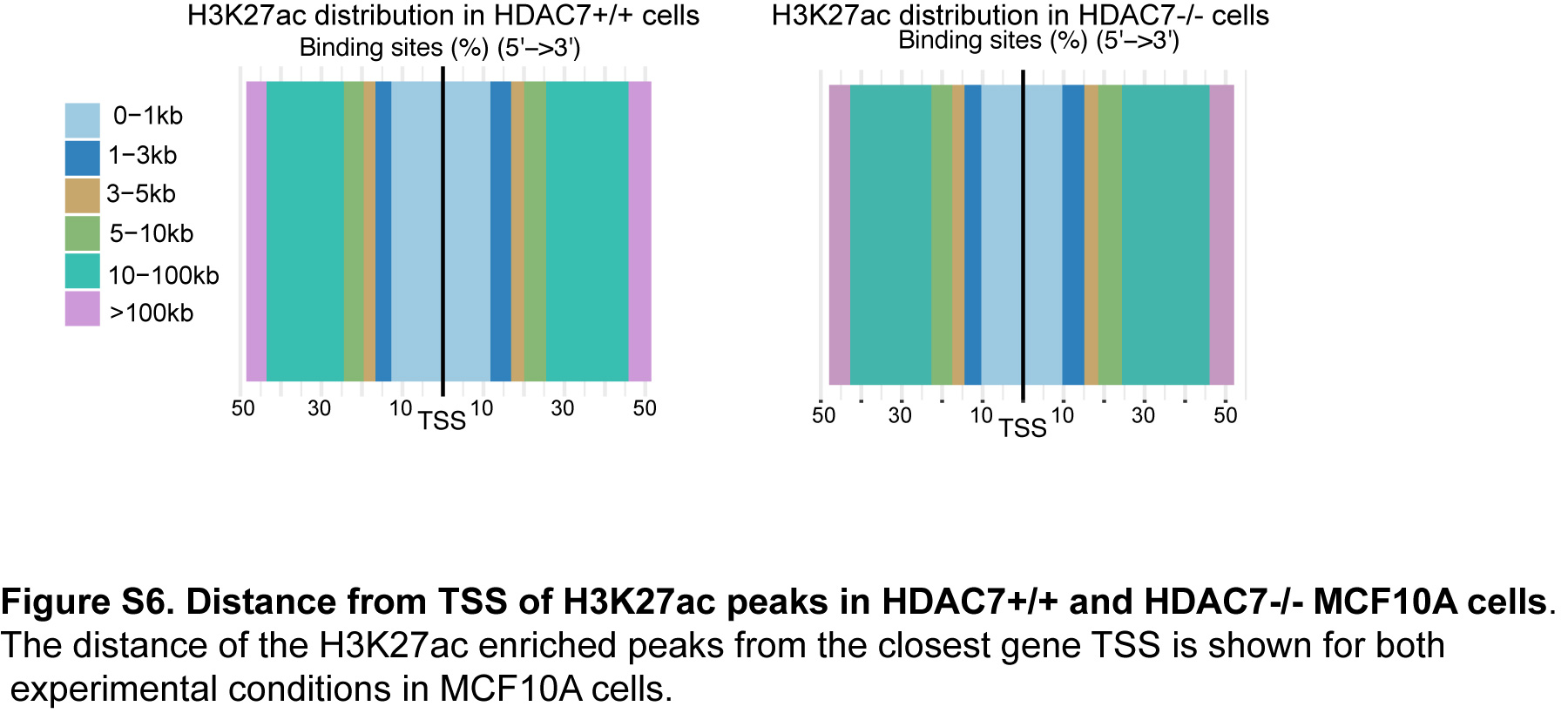

Supplement: Supplementary file 6 — Fig. S6. Distance from TSS of H3K27ac peaks in HDAC7 +/+ and HDAC7 −/− MCF10A cells. [file MOL2-13-1651-s006.jpg]

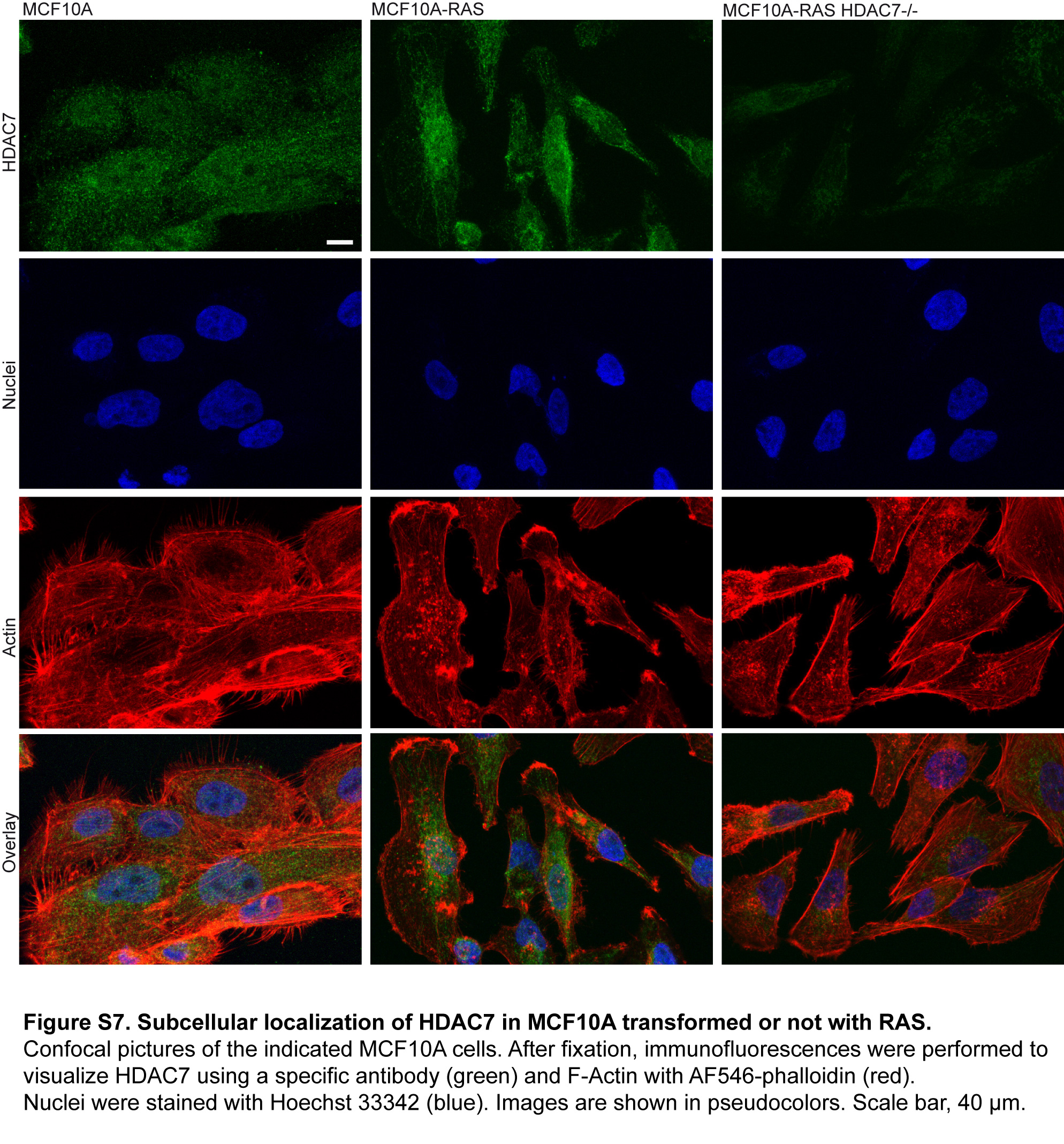

Supplement: Supplementary file 7 — Fig. S7. Subcellular localization of HDAC7 in MCF10A transformed or not with RAS. [file MOL2-13-1651-s007.jpg]
